# Asymmetric Aldol Reactions of α,β-Unsaturated Ketoester Substrates Catalyzed by Chiral Diamines

**DOI:** 10.3390/molecules16053778

**Published:** 2011-05-04

**Authors:** Sha-Sha Kan, Jian-Zhen Li, Cheng-Yan Ni, Quan-Zhong Liu, Tai-Ran Kang

**Affiliations:** Laboratory of Chemical Synthesis and Pollution Control, College of Chemistry and Chemical Engineering, China West Normal University, Nanchong 637009, Sichuan, China

**Keywords:** chiral diamine, enantioselectivity, aldol reaction, configuration

## Abstract

Highly efficient asymmetric aldol reactions between α,β-unsaturated keto esters and acyclic ketones catalyzed by chiral diamines are reported. The corresponding products were obtained in excellent yields with excellent enantioselectivities. The absolute configuration for the product was determined by X-ray analysis. A variety of substrates were tolerable in the present catalytic system.

## 1. Introduction

**Figure 1:**
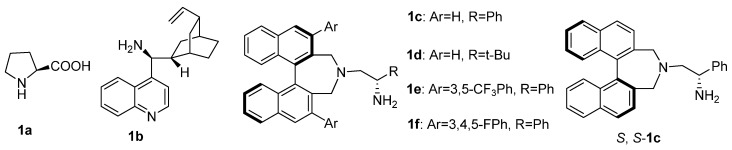
The catalysts surveyed in the work.

The asymmetric aldol reaction affording the corresponding β-hydroxy carbonyl compounds represents one of the most important methods for C-C bond formation [1,2,3,4,5,6,7,8,9,10,11,12,13,14,15,16]. This transformation has been successfully performed using Lewis acids as catalysts when activated ketones were used as substrates [17]. Recently direct asymmetric aldol reactions were also realized using organocatalytic methods. The organocatalytic aldol reaction has defined the current state of art in asymmetric synthesis and received great attention from the chemical community tahks to the facile preparation of the catalysts, environmentally benign features and mild reaction conditions. Proline and its derivatives, namely the prolinamides, have been found to be versatile catalysts, while chiral amines such as cinchona derived primary amines, 1,2-diaminohexane derivatives and other amines with primary-tertiary amine structure have also been evaluated in direct aldol reactions and significant progress has been achieved [18,19,20,21,22,23,24,25,26,27,28,29,30,31,32,33,34,35]. Despite the great success achieved in the area, some major challenges still remain. One of the most desirable goals in organic chemistry is the catalytic asymmetric assembly of simple and readily available precursor molecules into structurally complex products. In this context, α,β-unsaturated ketoesters have been used for the construction of functionally diverse organic compounds [36,37,38,39]. α,β-Unsaturated ketoesters should be a suitable aldol acceptor candidates. In fact, Tang has reported an enantioselective construction of bicyclic molecules via a Michael-aldol reaction using α,β-unsaturated ketoesters as starting material [40]. However, while acyclic ketones such as acetone provided acyclic products in excellent yields (99%), enantioselectivities were extremely low (14% *ee*). In 2008, Zhao reported an enantioselective aldol reaction between α,β-unsaturated ketoesters and ketones [41]. Although cyclic ketones afforded excellent yields and stereoslectivities, in case of acyclic ketones like acetone only a 45% *ee* was obtained with the reported procedure. However, the methodology provides a rapid access to a functionalities diversed product that are amenable to diverse transformations. To our knowledge, the aldol reaction between α,β-unsaturated ketoesters and cyclic ketones remained elusive. Recently we reported a highly efficient asymmetric aldol reaction between cyclic ketones and aromatic aldehydes catalyzed by cinchona derived amines [42]. We developed a chiral amine **1c** featuring a tertiary-primary amine motif and evaluated in the direct aldol reaction [43]. We surmised that structure **1** might be efficient catalyst in the direct aldol reactions between α,β-unsaturated ketoesters and ketones (Figure 1). Herein we discribed our work toward this effort.

## 2. Result and Discussion

Initially, the reaction between an α,β-unsaturated ketoester and acetone was chosen as the model reaction, and various catalysts including proline, cinchona alkaloid derived amine **1b** and the amines **1c** -**1f** developed by us were surveyed in the reaction. 

An α,β-unsaturated ketoester has two reaction sites, so the enamine formed from acetone and catalysts can attack either the β-position or the carbonyl position of the ketoester. In the first case, the Michael product may undergo a further aldol reaction forming a cyclic product. However, all the catalysts surveyed in the work ubiquitously formed the aldol product **4**. The results are summarized in Table 1.

In the initial investigation, **2a** reacted smoothly with acetone at room temperature in the presence of proline (10 mol%) affording the corresponding product in excellent yield albeit with low enantioselectivity (62% yield, 35% *ee*, Table 1, entry 1). Cinchona alkaloid-derived amine **1b** gave excellent yield (95% yield) with good enantioselectivity (75% ee), but a long reaction time was required (36 h). The catalyst **1c** which shows highly efficiency in the reaction of ketoesters and acetone afforded an almost quantitative yield (97% yield) and almost the same enantioselectivity (74% *ee*) in shorter reaction times, as the reaction proceeded to completion in 12 h. The catalyst **1c** was thus much more reactive than **1b**. Other catalysts **1d**-**1e** provided less satisfactory results compared to **1c**. Considering the reactivity of the catalystd, **1c** was chosen for further investigation. Acid screening found that TFA was the best candidate (Table 1, entries 3). The best ratio of catalyst to acid was found to be 1:1.5 (Table 1, entries 3, 11–12). The amounts of catalyst used had a significant influence on the reaction outcome. For example, increasing the catalyst loading resulted in higher yield and enantioselectivity. Ten mol % of catalyst was optimal considering both the yield and enantioselectivity. 

Solvent screening found acetone was the best choice; other solvents provided enantioselectivities ranging between 27–74% *ee*. General speaking, aprotic solvents such as DMSO and DMF provided higher selection than other solvents used (Table 2, entries 1–9). Temperature had a significant effect on the reaction, for example, lowering the temperature to −20 °C resulted in a dramatic increase in enantioselectivity (91% *ee* at −20 °C *vs.* 83% *ee* at 25 °C, entry 12 *vs.* entry 10). Too low a temperature retarded the reaction, and at −40 °C the reaction preceded very slowly affording 83% yield after 5 days although no drop in enantioselectivity was observed.

The optimized protocol was then expanded to a wide variety of α,β-unsaturated ketoesters and the results are summarized in Table 3. Various ketoesters reacted with acetone in excellent yields and enantioselecitvity. 

A brief survey of the scope of the reaction with respect to ketoester structure was carried out. Various esters including substrates substituted by electron withdrawing and donating groups at benzene ring attached to the esters uniformly provided the aldol products in near quantitative yields and with excellent enantioselectivities. For example, the introduction of methyl-, fluoro-, chloro-, or methoxy- groups at the *para* position of the benzene ring appended to the α,β-unsaturated ketoesters did not significantly affect the reactivity and enantioselectivity of the reaction (Table 2, entries 2–5). The steric hindrance in substrates had almost no effect on the stereoselectivity. *ortho*-Substituted esters such as both **2g** and **2h** afforded excellent yields and enantioselectivity. The results thus demonstrated good tolerance of substrates. 

**Figure 2:**
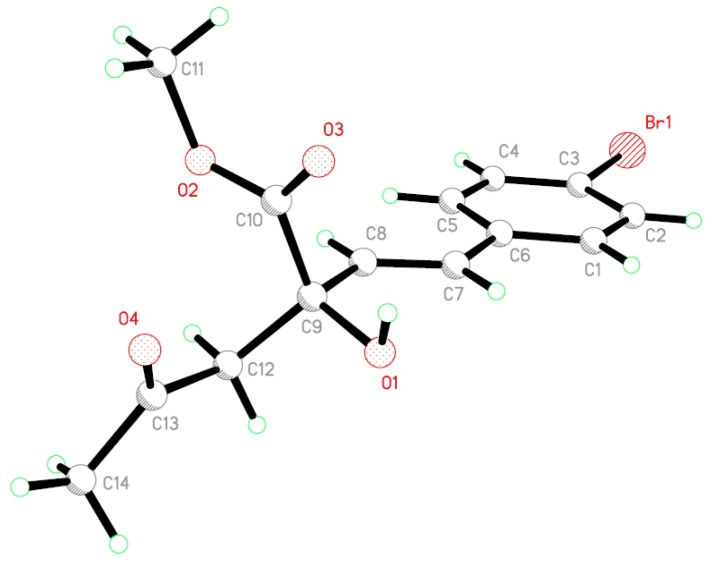
X-ray crystal structure of **4f** [45].

The absolute configuration of **4f** was determined to be *R* by X-ray diffraction of a single crystal formed in a mixed solvent of ethyl acetate in hexane (Figure 2). While this manuscript was in preparation Chan *et al*. reported an enantioselective aldol reaction using the same substrates catalyzed by cinchonine derived amine. The absolute configuration of **4f** was further verified to be *R* by the comparison of the retention time reported [44].

**Scheme 2:**
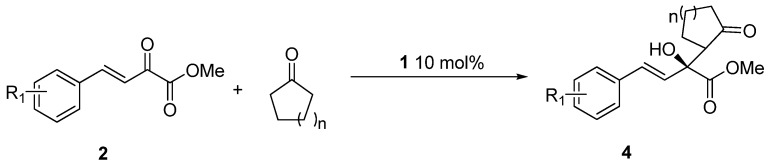
Reactions between α,β-unsaturated ketoester and cyclic ketones.

Cyclic ketones can also be used in this procedure. It should be noted that only aldol adducts were obtained with excellent enantioslectivity, although low diastereoselectivities were observed under our conditions and no bicyclic products were detected (Scheme 2). This was contrary to the procedure using prolinamides as catalysts [40].

## 3. Experimental

### 3.1. General

Unless otherwise noted, material were purchased from commercial suppliers and used without further purification. Acetone, cyclohexanone and cyclopentanone were freshly distilled. Flash column chromatography was performed using 200–300 mesh silica gel. ^1^HNMR spectra and ^13^CNMR spectra were recorded on a Bruker AVANCE-400 spectrophotometer. Chiral HPLC was performed on Shimadzu LC-20A with chiral columns (Chirapak AD-H and AS-H columns, Daicel Chemical Ind., Ltd.).

### 3.2. General Synthetic Pordecure for Aldol Reactions between α,β-unsaturated Ketoesters and Ketones

A solution of **1c** (0.01 mmol), TFA (0.015 mmol) and α,β-unsaturated ketoester **2a** (0.1 mmol) in acetone (1 mL) was stirred for about 24 h till TLC shows the disappearance of the ester. The reaction was quenched with saturated ammonium chloride solution (10 mL) and extracted with dichloromethane (3 × 15 mL). The combined organic layer was dried (anhydrous Na2SO4) and concentrated in vacuo. The crude product was submitted to column chromatography purification [petroleum ether-ethyl acetate = 8:1 (vv)], and the corresponding product **4a** was obtained in 95% yield. ^1^H-NMR (400 MHz, CDCl_3_): δ (ppm) 7.36–7.39 (m, 2H), 7.30–7.34 (m, 2H), 7.28–7.26 (m, 1H), 6.86 (d, *J* =15.8 Hz, 1H), 6.16 (d, *J* = 15.8 Hz, 1H), 4.01 (s, 1H), 3.81 (s, 3H), 3.27 (d, *J* =17.4 Hz, 1H), 2.93 (d, *J* =17.4 Hz, 1H), 2.20 (s, 3H). ^13^C-NMR (100 MHz, CDCl_3_): δ 206.7, 174.1, 135.8, 130.7, 128.5, 128.1, 127.9, 126.6, 75.1, 53.1, 51.6, 30.5. [α]_D_^25^ = +37.8, (C = 0.24). MS Exact mass calcd. for C_14_H_16_O_4_Na^+^: 271.0946, Found: 271.0950.

## 4. Conclusions

In summary, we have demonstrated that enantioselective aldol reactions between α,β-unsaturated ketoesters and acetone can be successfully realized in the presence of chiral diamines and Bronsted acids. Only aldol adducts were formed and no Michael-aldol products were detected. 

## Figures and Tables

**Scheme 1:**
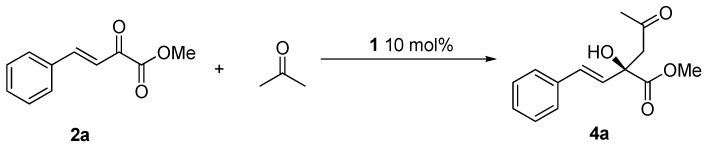
Reaction between α,β-unsaturated keto ester **2a** and acetone.

n = 1, 98% yield, dr = 1:1, 92% and 74% *ee*; n = 2, 97% yield, dr = 3:1, 94% and 7% *ee*.

**Table 1:**
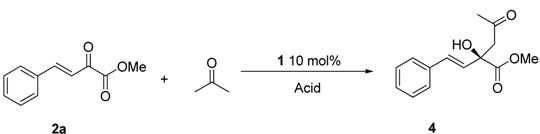
The reaction between α,β-unsaturated keto ester and acetone.^a^

Entry	Cat.	Additive	Time	Yield ^b^	Ee ^c^
1	**1a**	-	48	62	35
2	**1b**	TFA	36	98	75
3	**1c**	TFA	12	97	74
4	**1d**	TFA	12	94	54
5	**1e**	TFA	20	88	30
6	**1f**	TFA	25	96	56
7	**1c**	HAc	12	97	25
8	**1c**	PhCO_2_H	12	97	19
9	**1c**	TsOH	42	50	79
10	**1c**	TfOH	21	57	82
11 ^d^	**1c**	TFA	12	97	78
12 ^e^	**1c**	TFA	12	95	83
13 ^f^	***S,S*-1c**	TFA	18	86	71
14 ^g^	**1c**	TFA	10	87	78
15 ^h^	**1c**	TFA	10	95	79

^a)^ Unless otherwise stated, the reaction was carried out with 0.1 mmol of α,β-unsaturated keto- ester in the presence of 10 mol% of catalyst at room temperature with the combination of 20 mol% TFA using acetone as solvent; ^b)^ isolated yields; ^c)^ optical purity was determined using chiral HPLC; ^d)^ 10 mol% of TFA used; ^e)^ 15 mol% of TFA used; ^f)^ *S,S*-**1c** was used as catalyst under identical conditions of entry 12; ^g)^ 5 mol% of catalyst 1c in combination with 7.5 mol% TFA was used; ^h)^ 20 mol% of catalyst used.

**Table 2:**
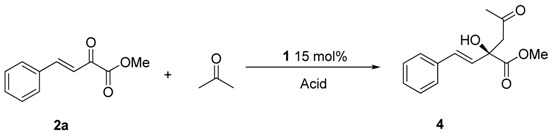
Solvent screening. ^a^

Entry	Solvent	Time	Yield	Ee
1	CH_2_Cl_2_	19	86	65
2	Toluene	19	85	67
3	THF	23	93	59
4	CH_3_CN	19	90	59
5	DMSO	19	95	74
6	Et_2_O	18	96	52
7	DMF	18	96	68
8	CHCl_3_	18	93	27
9	CH_3_CH_2_OH	18	89	53
10	acetone	12	95	83
11^b^	acetone	21	97	85
12^c^	acetone	18	95	91
13^d^	acetone	120	83	91

^a^ Unless otherwise stated, the reaction was carried out with 0.1 mmol of α,β-unsaturated ketoester in the presence of 10 mol% of catalyst at room temperature with the combination of 15 mol% TFA. ^b^ Reaction carried out at 0 °C; ^c^ Reaction carried at −20 °C; ^d^ Reaction carried at −40 °C.

**Table 3:**
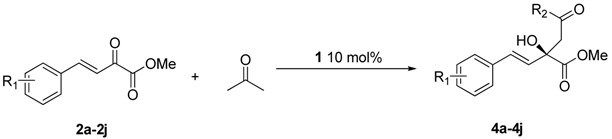
The reactions between α,β-unsaturated ketoesters and ketone.^a^

Entry	Product	R^1^	Yield ^b^	Ee ^c^
1	**4a**	H	95	91
2	**4b**	*p*-CH_3_	96	90
3	**4c**	*p*-NO_2_	91	96
4	**4d**	*p*-OMe	95	93
5	**4e**	*p*-F	93	92
6	**4f**	*p*-Br	96	94
7	**4g**	*p*-Cl	91	92
8	**4h**	*p*-CN	96	95
9	**4i**	*m*-CH_3_	86	92
10	**4j**	*m*-OMe	91	92

^a^ Unless otherwise stated, the reaction was carried out with 0.1 mmol of α,β-unsaturated ketoester in the presence of 10 mol% of catalyst using acetone as solvent; ^b^ isolated yields; ^c^ optical purity was determined using chiral HPLC.
